# QUALITY OF LIFE AND HEALTH SELF-PERCEPTION IN CHILDREN WITH POOR SCHOOL PERFORMANCE

**DOI:** 10.1590/1984-0462/;2017;35;4;00009

**Published:** 2017

**Authors:** Bárbara Antunes Rezende, Stela Maris Aguiar Lemos, Adriane Mesquita de Medeiros

**Affiliations:** a Universidade Federal de Minas Gerais, Belo Horizonte, MG, Brasil.

**Keywords:** Quality of life, Underachievement, Self-concept, Child, Speech-language pathology, Qualidade de vida, Baixo rendimento escolar, Autoimagem, Criança, Fonoaudiologia

## Abstract

**Objective:**

To examine the association between quality of life and health self-perception of children with poor school performance, considering sociodemographic factors.

**Methods:**

An analytical, observational, cross-sectional study was conducted with 99 children aged 7 to 12 years receiving specialized educational assistance. Parents and legal guardians answered questions concerning the sociodemographic profile. For an assessment of the quality of life and proposed domains (autonomy, functioning, leisure, and family), the children completed the *Autoquestionnarie Qualité de Vie Enfant Imagé* (AUQEI) and answered a question concerning their self-perceived health. Data were analyzed using multiple linear regression, considering a 5% significance level.

**Results:**

Among the evaluated children, 69 (69.7%) male participants with mean age of 8.7±1.5, 27% self-assessed their health status as poor/very poor, and 36.4% of the children reported having impaired quality of life. As for the domains assessed by AUQEI, there was statistical significance in the associations between family with age, autonomy with economic classification, and leisure and functioning with self-perceived health.

**Conclusions:**

The quality of life of children with academic underachievement is associated with their health self-perception and sociodemographic characteristics.

## RESUMO

**Objetivo:**

Verificar a associação entre qualidade de vida e autopercepção de saúde em crianças com mau desempenho escolar, considerando fatores sociodemográficos.

**Métodos:**

Estudo observacional analítico transversal com 99 crianças de 7 a 12 anos, participantes dos Atendimentos Educacionais Especializados. Os responsáveis responderam a questões sobre aspectos sociodemográficos. Para a avaliar a qualidade de vida e os domínios propostos (autonomia, funções, lazer e família) as crianças responderam ao *Autoquestionnarie Qualité de Vie Enfant Imagé* (AUQEI) e a uma questão referente à autopercepção de saúde. A análise de dados foi realizada por meio da regressão linear múltipla, considerando nível de significância de 5%.

**Resultados:**

Das crianças avaliadas, 69 (69,7%) eram do sexo masculino, com média de idade de 8,7±1,5. Do total, 27% delas autoavaliaram a saúde como ruim/muito ruim e 36,4% referiram ter qualidade de vida prejudicada. Quanto aos domínios avaliados pelo AUQEI, houve significância estatística dos domínios família e idade, autonomia e classificação econômica, lazer e funções em relação à autopercepção de saúde.

**Conclusões:**

A qualidade de vida de crianças com mau desempenho escolar está associada à autopercepção de saúde e a características sociodemográficas.

## INTRODUCTION

Poor school performance can be defined as academic achievement below the expected for a child’s chronological age, cognitive skills, and level of education.^1^ It can be one of the manifestations of a learning disorder or difficulty.^2^

Underachievement in school gives rise to limitations that could lead to low self-esteem and problems with acceptance by peers. Being less competent increases the feeling of incapacity and, in turn, contributes to continuing underachievement.^3^ Poor academic performance related to language impairment are possible contributing factors to the risk of social exclusion.^4^ In this context, children with poor academic performance are exposed to a number of factors that could impact their quality of life.

The term “quality of life” (QoL) is subjective and involves personal concepts of values, competencies, satisfaction, and well-being. The construct includes a variety of conditions that could affect the individuals’ perception, feelings and behaviors related to their daily-life functioning, including - but not limited to - their health status. The major characteristics linked to that concept are subjectivity and multi-dimensionality.^5^ ^,^ ^6^

Studies addressing the QoL of children have been more frequent in recent years, including those concerning disorders marked by functional impairment.^7^ ^,^ ^8^ ^,^ ^9^ The interest in investigating the QoL of children diagnosed with a learning disorder has been noted in other countries, such as Austria, India, and Greece.^10^ ^,^ ^11^ ^,^ ^12^ ^,^ ^13^ In Brazil, however, the knowledge on the QoL of children with academic underachievement is still scarce.

As from 1947, the definition of health according to the World Health Organization has expanded from absence of disease alone to a broad concept that can change in consonance with life’s perspectives and be related to the individuals’ sense of well-being.^14^ Health self-perception is considered a global index of health and encompasses physical, cognitive, and emotional dimensions. In addition, such perception relies on knowledge of health and disease that changes with life experience and social and cultural norms.^15^ ^,^ ^16^ Because such data are easily obtainable (typically elicited with a single question) and highly relevant, the assessment of children’s self-perception of health has received increasing attention in scientific studies. In light of the above, the aim of the present study was to investigate the association between QoL and health self-perception considering sociodemographic factors of children aged 7 to 12 years with poor school performance.

## METHOD

This analytical, observational, cross-sectional study investigated a probability sample (simple random sampling method) in children aged 7 to 12 years with poor school performance. All the children were participants of the specialized educational care in a country town in the state of Minas Gerais, Southeast Brazil.^17^ This specialized educational care was established by the Ministry of Education in order to complement or supplement the student’s training according to the specific needs of the children.^17^

The schoolchildren and their parents or guardians were invited to participate in the study; those who agreed provided a written informed consent. Participants were excluded if they did not complete the evaluation or had evidence or history of neurological, cognitive, and/or psychiatric alterations.

No estimates for expected percentages of impact on QoL, either general or domain-specific, were available to allow for sample size calculation. In view of that, the percentage was assumed to be 50% - number that maximizes the sample size. For the current study, 9% of sampling error and a 95% confidence interval were considered. A final sample of 90 children was estimated, considering the sampling universe of 617 students who were assisted by the specialized educational service in 2013.

This project was reviewed and approved by the UFMG Research Ethics Committee under No. CAAE 18683013.6.0000.5149.

The parents or legal guardians were interviewed and answered sociodemographic questions, including the socioeconomic profile according to the Brazilian Economic Classification Criteria (BECC).^18^ This classification considers the possession of goods and the level of education of the head of the household. The classes range from A1 to E, being A the class with the greatest purchasing power, and E, the lowest.

For assessing the children’s QoL, they completed the *Autoquestionnarie Qualité de Vie Enfant Imagé* (AUQEI) developed by Manificat and Dozart in 1997^19^ and validated for the Portuguese language by Assumpção Jr. et al. in 2000.^20^ This tool was chosen due to the fact that, in addition to being validated for Brazilian children, it presents the option of visual response, since the sample consisted of children with low academic achievement with possible impairment in reading and in comprehension skills.

The AUQEI is intended to assess an individual’s subjective well-being on the premise that developing individuals are capable of expressing themselves with regard to their subjectivity. The questionnaire relies on the perspective of the child’s satisfaction as identified in pictures (faces expressing different moods) associated with various domains of living, and comprises 26 questions that comprehend family relations, social relations, school activities, and health status.^20^ In the Portuguese validation study,^20^ children read the sentences with the support of pictures. In this research, there was an adaptation and the investigator read the sentences and asked each child to indicate the face expressing the feeling evoked by each situation presented. Previously, the child was requested to talk about one of his/her life experiences related to each answer chosen.

The questions were categorized into four domains:

1. *Functioning -* questions relating to activities in school, during meals, when going to bed, and visiting the doctor’s office (questions 1, 2, 4, 5, and 8) - Example: “(5) Tell me how you feel in the classroom”;
2. *Family -* questions addressing the child’s concept of his/her parents and of self (questions 3, 10, 13, 16, and 18) - Example: “(10) Tell me how you feel when you think of your father”;
3. *Leisure -* questions related to vacations, birthday, and relationship with the grandparents (questions 11, 21, and 25) - Example: “(21) Tell me how you feel during the holidays”;
4. *Autonomy -* questions concerning the child’s independence, relationship with peers, and academic performance (questions 15, 19, 23, and 24) - Example “(15) Tell me how you feel when you play alone”.

Questions 6, 7, 9, 12, 14, 17, 20, 22, and 26 were not included among the four domains; they have individual relevance as they represent separate domains. This division of domains was based on the original study for the design of the questionnaire.^19^ Scoring was established as follows: very unhappy = 0; unhappy = 1 point; happy = 2 points, and very happy = 3 points, with a maximum score of 78 points. The higher the score, the better the child’s judgment of his/her QoL. The instrument suggests a cutoff point of 48 points, meaning that those kids with total score lower than 48 have impaired QoL.^20^

After questionnaire completion, children’s overall health was assessed as follows: “In general, would you say that your health is: very good, good, fair, poor, or very poor?”.

For data analysis, the responses obtained with the instruments were organized, entered in a database and re-checked. First, a descriptive analysis was performed and reported by frequency, for categorical variables, central tendency measures and dispersion for continuous variables. Univariate and multiple linear regression analyses were conducted to determine the association between the total QoL score with the domains assessed by the instrument (autonomy, leisure, functioning, and family) and with health self-perception, adjusted for age, sex, and economic class. Statistical significance was set at 5%. The analyses were performed using the STATA software (Stata Corporation, College Station, Texas), version 13.0.

## RESULTS

In total, 99 children participated in the study, with 69 (69.7%) male participants and mean age of 8.7±1.5. Most participants were ranked in economic class C (64.6%) and 27% self-assessed their health status as poor/very poor (Table 1). The average total AUQEI score was 50.4 points, and 36.4% of the children reported having impaired quality of life.

**Table 1:** Descriptive analysis of sociodemographic characteristics of children with poor school performance.

	n (%)
Age	8.7± 1.5*
Sex	
Male	69 (69.7)
Female	30 (30.3)
Economic Classification	
A/B	19 (19.8)
C	64 (66.7)
D	13 (13.5)
Health self-perception	
Very good/Good	30 (30.3)
Fair	42 (42.4)
Poor/Very poor	27 (27.3)

*Mean/Standard Deviation.

An individual analysis of each instrument domain showed the highest mean scores for leisure, family, and functioning (7.7±1.2; 11.1±2.1; 9.6±2.3, respectively). Considering that the higher the mean, the better the QoL evaluation, the leisure and family domains were the ones most positively judged, whereas autonomy had the most negative evaluation (Table 2).

**Table 2:** Distribution of scores overall and by domain according to the AUQEI.

Characteristics	Minimum	Maximum	Range	Median	Mean	Standard Deviation
Quality of life	34	69	0-78	50	50.4	6.7
Autonomy	2	11	0-12	5	5.1	1.7
Leisure	4	9	0-9	8	7.7	1.2
Functioning	4	15	0-15	10	9.6	2.3
Family	6	15	0-15	11	11.1	2.1

Table 3 presents the univariate linear regression model, being possible to observe statistically significant association (p≤0.05) between health self-perception and the following areas: leisure, functions and family. Also, there was significant association between age and the family domain; between the economic classification with the autonomy domain and with the total score of the quality-of-life instrument.

**Table 3:** Univariate linear regression model for the AUQEI domains *versus* sociodemographic characteristics of children with poor school performance.

	Total QoL Score		Autonomy		Leisure		Functioning		Family	
	Coef.	p- value	Coef.	p- value	Coef.	p- value	Coef.	p- value	Coef.	p- value
Age	-0.56	0.2	-0.008	0.94	-0.069	0.36	-0.118	0.43	-0.337	0.01*
Sex										
Male	1		1		1		1		1	
Female	-0.823	0.57	0.002	0.99	-0.724	0.77	0.459	0.35	-0.127	0.78
Economic Classification										
A/B	1		1		1		1		1	
C	-2.875	0.09	-0.621	0.16	-0.359	0.23	-0.15	0.79	-0.424	0.42
D	-5.615	0.01	-1.238	0.04	-0.384	0.36	-0.976	0.23	-1.419	0.06
Health self-perception										
Very good/Good	1		1		1		1		1	
Fair	-2	0.25	-0.277	0.54	-0.013	0.96	-0.791	0.16	-1.006	0.05
Poor/Very poor	-3.5	0.14	0.166	0.78	-0.791	0.05	-2.458	<0.01	-0.511	0.48

QoL: quality of life; Coef.: coefficient.

The multiple linear regression model outcomes are shown in Table 4. They reveal statistically significant associations of leisure and functioning with self-perception of health; family domain with age; and autonomy domain with economic classification.

**Table 4:** Multiple linear regression model for the AUQEI domains *versus* sociodemographic characteristics of children with poor school performance.

	Total QoL score			Autonomy			Leisure			Functioning			Family			
	Coef.	p- value	R^**2**^	Coef.	p- value	R^**2**^	Coef.	p- value	R^**2**^	Coef.	p- value	R^**2**^	Coef.	p- value	R^**2**^
Age	-0.558	0.23	0.1	0.06	0.62	0.05	-0.107	0.19	0.07	-0.203	0.19	0.14	-0.33	0.02*	0.1
Sex															
Male	1			1			1			1			1		
Female	-0.386	0.79		0.087	0.82		-0.024	0.92		-0.63	0.2		0.007	0.98	
Economic Classification															
A/B	1			1			1			1			1		
C	-2.586	0.13		-0.687	0.13		-0.274	0.36		0.004	0.99		-0.273	0.6	
D	-4.418	0.07		-1.357	0.04		-0.155	0.72		-0.564	0.48		-0.909	0.23	
Health self-perception															
Very good/Good	1			1			1			1			1		
Fair	-2.347	0.2		-0.321	0.5		-0.074	0.81		-0.652	0.28		-1.039	0.06	
Poor/Very poor	-3.676	0.13		0.369	0.56		-0.907	0.03		-2.742	<0.01		-0.84	0.264	

QoL: quality of life; Coef.: coefficient; R^2^: coefficient of determination.

## DISCUSSION

This study showed that poor school performance have a negative impact on quality of life and health perception for children. Studies with children who have had a recent diagnosis of learning disabilities found similar results, but using other tools to assess the quality of life.^11^ ^,^ ^12^ There are no studies about quality of life of children with poor school performance using the AUQEI. However, studies were found evaluating children with leukemia^21^ and cystic fibrosis with AUQUEI,^22^ and the prevalence of impaired quality of life was 15% and 25%. It is plausible to assume that children who have passed or have experienced serious diseases value situations and areas investigated by the AUQEI, which justify a better evaluation of the construct “quality of life”.

The analysis of the AUQEI instrument by domains showed that children had greater satisfaction with tasks related to leisure and family relationships, whereas situations demanding autonomy were the least satisfying ones. As cited before, no studies were found in the literature using the AUQEI to investigate the QoL of children with poor academic performance. However, the AUQEI was used to evaluate children with neoplasms^23^ and stomas,^24^ and showed that the autonomy domain was judged more negatively by children, whereas family had the most favorable assessment, which corroborates the findings of the present study.

The multiple linear regression analysis showed statistical significance in the association between the family domain and age. As age increases, the means for a child’s perception of family relations decrease, indicating a QoL deterioration in that domain. The literature indicates that family is a very relevant social institution, with the potential to influence the subjective well-being of children.^25^ However, a study from India evaluated 150 children with mean age of 12.2 years who had poor academic achievement and found no such association.^11^ The findings of the current research support the hypothesis that older children can better characterize their social and family context, which corroborates a Brazilian study in which older institutionalized children with low academic achievement had inferior means on the Multi-Dimensional Life Satisfaction Scale.^26^

The sex variable showed no statistically significant association with the domains investigated. The literature corroborates this finding,^11^ although one study found that girls had worse scores for “social exclusion” and “overall health”.^12^

The autonomy domain was significantly associated with economic classification, as children in economic class D scored lower in the autonomy domain compared to those in classes A to C. The correlation coefficient demonstrated a tendency toward lower means in the autonomy domain with lower ranking in the BECC. Socioeconomic status is known to affect an individual’s perception of the social setting. A review study showed that parents in lower socioeconomic classes prioritize conformity values; as a result, they tend to apply more coercive parenting practices, including the use of force and power assertion, whereas those in a higher socioeconomic class value autonomy and self-control, and typically use inductive discipline methods that favor the children’s reflection on the situations. However, this should be regarded with caution, since not only families with low socioeconomic status adopt coercive practices.^27^ Besides, these factors were not measured in this study and cannot be compared.

A study using the PedsQL questionnaire found that children from families with low socioeconomic status had poor QoL, including the psychosocial health scale and the dimensions of emotional, social, and academic functioning.^10^ Another study showed that autonomy, as assessed by the DISABKIDS instrument, was also associated with lower economic status.^12^ It should be noted that both studies were conducted with children who had poor academic achievement, which suggests that the independence of those children could reflect on their perception of QoL. The children from households with lower purchasing power are likely faced with fewer situations demanding greater autonomy, and may, therefore, have little power of decision. In addition, they tend to spend more time alone. Thus, it would be logical to have better results; however, the effect may be contrary, because the kids in this situation may feel pressed, which justifies the worst results.

The leisure and functioning domains - which relate to activities that promote the children’s well-being and to the degree of satisfaction about their functional capacity, respectively - were significantly associated with self-perceived health. When self-perception was compared across the range from “very good” to “very poor” in the multiple linear regression model, mean reductions of 0.9 to 2.7 were found for the leisure and functioning domains, respectively; this shows a greater downward trend in satisfaction with the activities of daily living (functioning). The prevalence of a negative health self-perception was similar to that observed in a study conducted in a Southern Brazilian city: 25.7% of 1,134 students aged 14 to 19 years had negative health self-perception.^28^ The authors of a study that included Brazilian, Canadian, Chinese, and Italian children investigated the self-perception of competencies of 1,534 schoolchildren with mean age of 12 years using the Self-Perception Profile for Children. The authors identified significant differences across the countries in the social and academic domains, and they demonstrated that negative perceptions of competencies may be associated with academic underachievement.^16^

The school experience plays a critical role in building the self-perception of children. No studies were found in the literature that investigated the association between children’s self-perception and their QoL. It is known that children with poor academic achievement may have a high risk of developing a negative self-concept.^29^ A Brazilian study evaluated 1,070 schoolchildren with the aim of investigating the self-perception of students in development areas connected to school performance; the results were statistically significant for the correlations between grade failure and parental level of education.^30^

The coefficient of determination was higher in the multiple linear regression model for the functioning domain: approximately 14% of the functional impairment can be explained by poorer health self-perception. The present study demonstrated that the children’s self-perceived health is related to their QoL, and that these concepts are likely formulated from the socioeconomic and cultural contexts in which those children are inserted. Thus, health and education professionals should evaluate children globally, in addition to their academic difficulties.

Importantly, caution is required when analyzing the results of this study, since the questionnaires assessing the QoL of children with poor school performance available in the literature are different from that administered in the present study, which complicates comparisons. Also, the perception of the parents on the QoL of their children should be addressed in further investigations. Due to the cross-sectional design of the present study, it is not possible to establish a causal relationship between the AUQEI domains and the aspects investigated.

In conclusion, the scores of QoL domains for children with poor performance at school are associated with worse health self-perception and sociodemographic characteristics. Children’s health, as perceived from their own perspective, can be an important index of their socio-cultural setting and value systems in relation to their goals, expectations, and interests.
